# Eradication of hepatitis C virus infection in kidney transplant recipients using direct‐acting antiviral therapy: Qatar experience

**DOI:** 10.1002/iid3.386

**Published:** 2020-12-02

**Authors:** Mohamad M. Alkadi, Essa A. Abuhelaiqa, Mostafa F. Elshirbeny, Ahmed F. Hamdi, Omar M. Fituri, Muhammad Asim, Saad R. Alkaabi, Moutaz F. Derbala, Mona E. Jarman, Adel M. Ashour, Awais Nauman, Yousuf K. Al Maslamani, Adeel A. Butt, Hassan A. Al‐Malki

**Affiliations:** ^1^ Division of Nephrology, Department of Medicine Hamad Medical Corporation Doha Qatar; ^2^ Weill Cornell Medical College in Qatar Doha Qatar; ^3^ Division of Gastroenterology, Department of Medicine Hamad Medical Corporation Doha Qatar; ^4^ Division of Transplantation Surgery, Department of Surgery Hamad Medical Corporation Doha Qatar; ^5^ Department of Medicine Hamad Medical Corporation Doha Qatar

**Keywords:** hepatitis C, immunosuppression, kidney function, kidney transplantation

## Abstract

**Introduction:**

Hepatitis C virus (HCV) infection has detrimental effects on patient and graft survival after kidney transplantation. In the pre‐direct‐acting antiviral (DAA) era, treatment of HCV infection was associated with low response rates, poor tolerance, and increased risk of allograft rejection. However, DAAs have revolutionized HCV treatment. The aims of this study were to determine the impact of DAA on the sustained virologic response (SVR), renal function, and calcineurin inhibitor (CNI) levels and assess the tolerability to treatment in kidney transplant recipients with HCV infection in Qatar.

**Methods:**

This retrospective study included the medical records of all kidney transplant recipients with confirmed HCV infection before January 1, 2020. All data were obtained from the patients’ electronic medical records; these included patient demographics; virologic responses to treatment; serum creatinine levels during treatment; urine protein to creatinine ratios and CNI levels before, during, and after treatment; and side effects related to DAA therapy.

**Results:**

A total of 27 kidney transplant recipients with HCV were identified, 23 of whom received DAA therapy. The length of treatment ranged from 12 to 24 weeks, and 52% of patients had HCV genotype 1 infection. The median log10 HCV RNA was 6.6 copies per milliliter. None of the patients had liver cirrhosis, and all of them achieved SVR. There was no statistically significant difference in the glomerular filtration rate before, during, and after treatment. Most patients had stable CNI trough levels during treatment and did not require dose adjustment.

**Conclusions:**

HCV infection was successfully eradicated by DAA therapy in kidney transplant recipients, with a 100% SVR rate. Moreover, DAA therapy was well‐tolerated, and kidney function remained stable without an increased risk of rejection. These results are expected to drive the eradication of hepatitis C from the entire country.

## Graphical abstract

Direct‐acting antivirals (DAAs) have revolutionized hepatitis C virus (HCV) treatment. The aims of this study were to determine the impact of DAA on the sustained virologic response (SVR), renal function, and calcineurin inhibitor (CNI) levels and assess the tolerability to treatment in kidney transplant recipients with HCV infection in Qatar. We believe that our study makes a significant contribution to the literature because, in line with the WHO vision, we were able to achieve the eradication of HCV infection among kidney transplant recipients in Qatar by administering DAA therapy, which yielded a complete treatment response in all recipients infected with HCV.

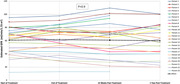

**Abbreviations**
AKI: acute kidney injury
ANOVA: analysis of variance
APRI: aspartate aminotransferase to platelet ratio index
CKD: chronic kidney disease
CNI: calcineurin inhibitor
CYP: cytochrome P
DAA: direct‐acting antiviral
DNA: deoxyribonucleic acid
eGFR: estimated glomerular filtration rate
ESRD: end‐stage renal disease
Fib‐4: fibrosis‐4
GHSS: global health section strategy
HBV: hepatitis B virus
HCV: hepatitis C virus
HIV: human immunodeficiency virus
HMC: Hamad Medical Corporation
KDIGO: kidney disease improving global outcomes
MoPH: Ministry of Public Health
PCR: polymerase chain reaction
PTDM: posttransplant diabetes mellitus
SVR: sustained virologic response
WHO: World Health Organization

## 1 INTRODUCTION

Chronic hepatitis C virus (HCV) infection is an important global health problem. The prevalence of HCV is significantly higher in hemodialysis and kidney transplant recipients than in the general population.^1^, ^2^, ^3^ The high risk of HCV infection in kidney transplant recipients might be acquired from organ donors or related to hemodialysis and frequent blood transfusions. A study showed that the HCV viral load increased to approximately 1.0–1.5 log10 IU/ml after renal transplantation and that pretransplantation HCV infection may be associated with an increased risk of liver disease and death after transplantation.^4^

Several studies have shown that HCV has a detrimental effect on both patient and graft survival after kidney transplantation.^5^, ^6^, ^7^, ^8^ These studies suggested that cirrhosis and other liver‐related complications are associated with shorter survival of HCV‐positive kidney transplant recipients. Given the high risk of liver disease progression and increased mortality in kidney transplant recipients with active HCV infection, HCV treatment is highly recommended for patients affected by chronic kidney disease (CKD), either before or after transplantation, as per the Kidney Disease, Improving Global Outcomes (KDIGO) guidelines.^9^ The initial KDIGO guideline published in 2008 provided recommendations for the prevention, diagnosis, and management of HCV in CKD patients.^10^ Since then, there have been major advances in HCV management, particularly with the advent of direct‐acting antiviral (DAA) therapy.

In the pre‐DAA era, there was limited use of interferon and ribavirin for the treatment of HCV infection in kidney transplant recipients because of the risk of allograft rejection and intolerance.^10^ Although international published data on DAAs in kidney transplant recipients are less abundant, the results appear as satisfactory as those observed for liver transplant recipients.^11^ The state of Qatar has only a 2% prevalence of HCV; however, the impact of infection is detrimental in the country because of late presentation.^12^ This led to the development of the Qatar National plan established by the Qatar Ministry of Public Health (MoPH) and Hamad Medical Corporation (HMC) for HCV control, which was approved by the Qatar government in December 2014. The aims of this study were to determine the impact of DAA therapy on sustained virologic response (SVR) rates, renal function, and calcineurin inhibitor (CNI) levels, and to assess the treatment tolerability and side effects, in kidney transplant recipients with positive HCV infection in Qatar.

## 2 MATERIALS AND METHODS

### 2.1 Study population

This retrospective study was conducted at HMC, the only healthcare facility that provides kidney transplantation care in the State of Qatar, and it was approved by our local institutional review board (RP 17039/17). Following the establishment of the Qatar National plan for HCV control, DAA therapy was introduced at HMC in 2015. At that time, we decided, in collaboration with the gastroenterology division, to eradicate HCV infection in all kidney transplant recipients living in Qatar. Patients who underwent transplantation before 2015 (*n* = 555) were screened with HCV polymerase chain reaction (PCR) if they were never serologically tested for HCV or had a positive HCV serology after kidney transplant. On the contrary, all transplantation patients from 2015 onwards (*n* = 314) were screened with only PCR.

We also began testing all patients who underwent kidney transplantation abroad at the time of their first visit to our kidney transplant clinics. Patients with positive HCV RNA as per PCR were immediately referred to the viral hepatitis clinic for further evaluation and management.

For the present study, we retrospectively studied all kidney transplant recipients with documented HCV infection after kidney transplantation. There were 23 patients who received DAA therapy, and 48% were treated more than 5 years after kidney transplantation. The length of treatment with DAA per‐protocol ranged between 12 and 24 weeks depending on the HCV genotype, HCV treatment history, and virologic response during treatment. For instance, patients with HCV genotype 3 required treatment for up to 24 weeks. Patients were tested for HCV PCR before treatment, at 4 weeks after starting DAA therapy, at the end of treatment, at 3 and 12 months after treatment completion and then annually. If HCV at 4 weeks after starting DAA therapy was detected, then HCV PCR was repeated every 2 weeks until it became negative. Patients were also tested if they had any change in the level of their hepatic transaminases during regular follow‐ups.

All data were obtained from the patients’ electronic medical records; these included patient demographics; virologic responses to treatment; serum creatinine levels during treatment; CNI levels before, during, and after treatment; and side effects related to DAA therapy. The last CNI trough level before DAA therapy initiation, all trough levels during treatment, and the first trough level after DAA therapy completion were reviewed. Renal function was assessed by measuring the estimated glomerular filtration rate (eGFR) measured at the start of DAA, at the end of DAA, and 3 months and 1 year after treatment completion. Serum creatinine values during the treatment period were also reviewed to determine the incidence of acute kidney injury (AKI).

### 2.2 Definitions

AKI was defined as an increase in the serum creatinine level of 26.5 μmol/L or more from baseline. eGFR was calculated from the serum creatinine level using the Chronic Kidney Disease Epidemiology Collaboration equation. Liver fibrosis was assessed using the fibrosis‐4 (Fib‐4) index and the aspartate aminotransferase to platelet ratio index (APRI). SVR was defined as the absence of HCV RNA in the blood 3 months after the completion of DAA therapy.

### 2.3 Statistical analysis

Data were summarized using frequency measures for categorical variables and mean and standard deviation (*SD*) values for continuous variables. One‐way analysis of variance (ANOVA) was used to determine *p* values for differences in serum creatinine levels before and during treatment and differences in CNI levels among the different time points.

## 3 RESULTS

### 3.1 Study population

**Figure 1:**
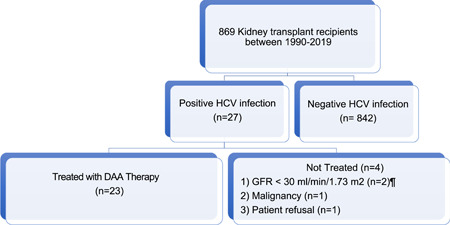
Study design for the assessment of kidney transplant recipients with positive hepatitis C viral infection. In total, 27 of 869 kidney transplant recipients had HCV infection. From these, 23 patients received DAA therapy. DAA, direct‐acting antiviral, GFR, glomerular filtration rate, HCV, hepatitis C virus. ^¶^All available DAA regimens before 2018 were sofosbuvir‐based

In total, 27 (3.1%) of 869 kidney recipients transplanted locally or abroad between 1990 and January 1, 2020 had HCV infection, and 23 of these patients received DAA therapy. The remaining four patients were not treated because of malignancy (*n* = 1), patient refusal (*n* = 1), or an eGFR of <30 ml/min/1.73 m^2^ (*n* = 2), as all available DAA regimens at our center until 2018 were sofosbuvir‐based. Both patients with eGFR < 30 ml/min/1.73 m^2^ progressed to end‐stage renal disease (ESRD) before 2018, while the other two patients died with a functioning graft before 2020. The study design is summarized in Figure 1.

The mean age of patients at the time of DAA treatment was 46.5 ± 11.6 years. Most patients were of Middle Eastern origin (*n* = 13; 57%). Three patients had both liver and kidney transplants. HCV infection before kidney transplantation was documented for 39% patients (*n* = 9). The median serum log10 HCV RNA before treatment initiation was 6.6 copies per milliliter. HCV 1a was the most common genotype (*n* = 10; 43%). None of the patients suffered from liver cirrhosis at the time of treatment, based on their calculated APRI and FIB4 scores (APRI > 1.5 or FIB4 > 3.25). Moreover, no patient was diagnosed with cryoglobulinemia. The baseline characteristics of the patients who received DAA therapy are summarized in Table 1.

**Table 1:** Baseline characteristics of kidney transplant patients infected with hepatitis C virus and treated with direct‐acting antiviral therapy

Baseline characteristics	*N* = 23
Year of transplant, *n* (%):	
1990–1999	7 (30)
2000–2009	1 (4)
2010–2019	15 (65)
Native kidney disease, *n* (%)	
Hypertensive nephrosclerosis	5 (22)
Diabetic nephropathy	4 (17)
Immunoglobulin A nephropathy	1 (4)
Focal segmental glomerulosclerosis	1 (4)
Polycystic kidney disease	1 (4)
Congenital renal disease/reflux nephropathy	3 (13)
Others/unknown	8 (35)
Age at transplant, years, mean (SD)	37.8 (12.7)
Male recipients, *n* (%)	17 (74)
Recipient ethnicity, *n* (%)	
Middle Eastern	13 (57)
Asian	7 (30)
African	3 (13)
Place of transplant, *n* (%)	
Middle East	12 (52)
Asia	9 (39)
United Kingdom	2 (9)
Donor type, *n* (%)	
Living related	13 (57)
Living unrelated	10 (43)
Liver transplant, *n* (%)	3 (13)
Maintenance immunosuppression, *n* (%)	
Tacrolimus + mycophenolate mofetil + steroids	11 (48)
Tacrolimus + mycophenolate mofetil	2 (9)
Cyclosporine + mycophenolate mofetil + steroids	5 (22)
Tacrolimus + azathioprine + steroids	4 (17)
Cyclosporine + azathioprine + steroids	1 (4)
Documented HCV infection pretransplant, *n* (%)	9 (39)
HCV genotype, *n* (%):	
1a	10 (43)
1b	2 (9)
3	4 (17)
4	7 (30)
HCV RNA log10 before DAA therapy, copies/ml, median (IQR)	6.6 (6.4–7.2)
Fibrosis scores before DAA therapy	
FIB‐4 Score, median (IQR)	0.9 (0.6–1.1)
APRI Score, median (IQR)	0.3 (0.2–0.4)
eGFR before DAA therapy, ml/min, median (IQR)	67.3 (44.1–73.5)
Age at DAA therapy, years, mean (SD)	46.5 (11.6)
Time from transplant to DAA initiation, years, median (IQR)	4.1 (2.3–17.8)
DAA therapy, *n* (%):	
Daclatasvir + sofosbuvir	13 (56)
Ledipasvir + sofosbuvir	8 (35)
Simeprevir + sofosbuvir	1 (4)
Elbasvir + grazoprevir	1 (4)
Duration of DAA therapy:	
12 weeks, *n* (%)	16 (70)
24 weeks, *n* (%)	7 (30)
Follow‐up after DAA therapy, months, median (IQR)	32.6 (23.1–41.3)

Abbreviations: APRI, aspartate aminotransferase to platelet ratio index; DAA, direct‐acting antiviral; eGFR, estimated glomerular filtration rate; FIB‐4, fibrosis‐4; HCV, hepatitis C virus; IQR, interquartile range; RNA, ribonucleic acid; *SD*, standard deviation.

### 3.2 Efficacy of DAA therapy

All patients with eGFR > 30 ml/min/1.73 m^2^ were treated with sofosbuvir‐based regimens; 13 received daclatasvir (57%), eight received ledipasvir (35%), and one received simeprevir (4%). There was only one patient with eGFR < 30 ml/min/1.73 m^2^ in 2018, and he received elbasvir and grazoprevir. The duration of DAA therapy ranged between 12 and 24 weeks depending on the HCV genotype, HCV treatment history, and virologic response during treatment. Three patients began receiving entecavir in addition to DAA therapy; one of them had concomitant HBV infection, while the other two only had HBV core antibodies, with negative HBV surface antigen and undetected HBV DNA. All patients had undetected serum HCV RNA within 8 weeks of receiving DAA therapy, regardless of the HCV genotype. All patients achieved SVR at 3 months after treatment. The median follow‐up duration after DAA therapy was 31.2 months (interquartile range [IQR]: 14.1–37.8), and no patient showed positive HCV PCR results during the follow‐up.

The median time for testing after completing therapy was 21.7 (13.0–31.5) months.

### 3.3 Effect of DAA therapy on renal function and CNI trough levels

**Figure 2:**
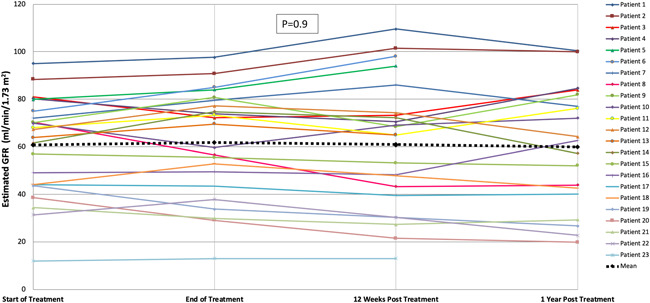
Renal function of kidney transplant recipients with positive hepatitis C virus infection treated with direct‐acting antiviral (DAA) therapy. The glomerular filtration rate (GFR) was estimated using the CKD‐EPI equation. Each line in the figure represents a patient. For example, patient number 1 had an estimated GFR of 95 ml/min/1.73 m^2^ at the start of DAA therapy, 98 ml/min/1.73 m^2^ at the end of treatment, 110 ml/min/1.73 m^2^ at 3 months, and 100 ml/min/1.73 m^2^ at 1 year after treatment completion. There is no 1‐year eGFR data for patients 5, 6, and 13 because they completed their treatment less than a year ago, at the time of preparing this figure. Patient 23 progressed to ESRD and died before reaching the 1‐year evaluation point. The dotted black line represents mean eGFR among the four evaluation points (start of treatment, end of treatment, and 12 weeks and 1 year after treatment completion); there is no statistically significant difference in eGFR (*p* = .9). CKD‐EPI, Chronic Kidney Disease Epidemiology Collaboration; ESRD, end‐stage renal disease

There was no statistically significant difference in eGFR among the four‐time points: The mean eGFR values at the start of DAA therapy, at the end of treatment, and at 3 months and 1 year after treatment completion were 60.7, 61.7, 60.9, and 59.9 ml/min/1.73 m^2^, respectively (*p* = .9; Figure 2). Three patients (13%) experienced an AKI episode during DAA therapy. The first patient had HCV genotype 3 and was receiving sofosbuvir/daclatasvir. Her serum creatinine increased from 154 to 250 μmol/L in the setting of urinary tract infection and returned to the baseline value in a few days. The second patient had HCV genotype 1b and was receiving sofosbuvir/simeprevir; his creatinine increased from 174 to 240 μmol/L, and his tacrolimus trough level was 17 ng/ml while taking azithromycin. The patient underwent kidney biopsy, which showed collapsing glomerulopathy with moderate tubular atrophy and interstitial fibrosis. The third patient had HCV genotype 1b and was receiving elbasvir/grazoprevir; his creatinine increased from 457 to 540 μmol/L, and he required temporary hemodialysis. However, his kidney function recovered and his eGFR value at the end of treatment was the same as that at the start of treatment. None of the 23 patients exhibited acute rejection during treatment.

**Figure 3:**
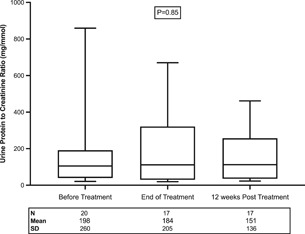
Effect of direct‐acting antiviral (DAA) therapy on proteinuria in kidney transplant recipients with hepatitis C virus infection. Box and whisker plot of urine protein to creatinine ratio at the start of DAA therapy at the end of treatment, and at 12 weeks after treatment completion. The mean protein to creatinine ratio was 198 ± 260, 184 ± 205, and 151 ± 136 mg/mmol, respectively; and there were no significant differences among the time points (*p* = .85)

Before starting DAA therapy, proteinuria was observed in 52% of patients (*n* = 12). The mean urine protein to creatinine ratio at the start of DAA therapy, at the end of treatment, and at 12 weeks after treatment completion were 198 ± 260, 184 ± 205, and 151 ± 136 mg/mmol, respectively and there were no significant differences among the time points (*p* = .85; Figure 3).

**Figure 4:**
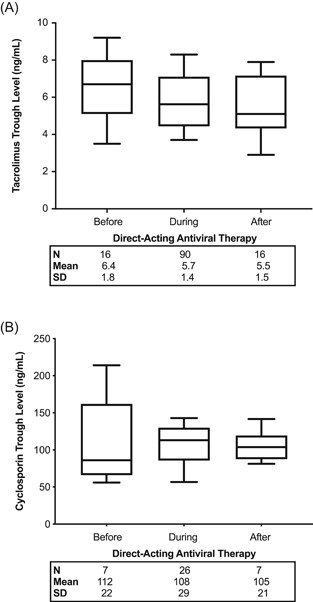
Effect of direct‐acting antiviral (DAA) therapy on calcineurin inhibitor trough levels in kidney transplant recipients with hepatitis C virus infection. Box and whisker plot of the mean (A) tacrolimus (*n* = 16) and (B) cyclosporine (*n* = 7) trough levels before, during, and after DAA therapy is shown. The mean time of calcineurin inhibitor (CNI) trough level before initiation of DAA, first trough level after initiation of DAA and first trough level after treatment completion was 18 ± 15, 21 ± 12, and 30 ± 23 days, respectively. The mean number of CNI trough levels obtained during DAA therapy was 5 ± 2 samples per patient

In terms of immunosuppression, all patients were maintained on CNI‐based regimens, with 16 receiving tacrolimus (70%) and seven receiving cyclosporine (30%). The mean time of CNI level before initiation of DAA, first level after initiation of DAA and first level after treatment completion was 18 ± 15, 21 ± 12, and 30 ± 23 days, respectively. CNI trough levels are summarized in Figure 4A,B. Most patients showed similar CNI levels before, during, and post‐DAA therapy and did not require dose adjustment. However, one and two patients required an increase and a decrease in the CNI dose during treatment, respectively (13%).

### 3.4 Side effects related to DAA therapy

DAA therapy was generally well‐tolerated by most patients. Seven of the 23 patients (30%) reported side effects. Fatigue was the most common side effect (*n* = 5), followed by insomnia (*n* = 4), nausea (*n* = 4), dizziness (*n* = 3), and diarrhea (*n* = 2). None of the patients reported headache, myalgia, cough, or skin rash during DAA therapy.

## 4 DISCUSSION

With the increasing global burden of HCV relative to that of other chronic infections such as HIV and tuberculosis, the United Nations has placed HCV treatment among its top priorities in the 2030 goals for sustainable development. Moreover, the World Health Organization (WHO) has outlined a global health section strategy (GHSS) on viral hepatitis for 2016–2021, in hopes to halt the transmission of this disease.^13^, ^14^ The ultimate goal of establishing a global vision and strategies is to eliminate viral hepatitis as a major public health threat in the distant future. Here we summarize our experience with the diagnosis and treatment of chronic HCV infection in kidney transplant recipients in Qatar. We observed 100% resolution of infection following DAA treatment, with all patients achieving SVR at 3 months after treatment. Thus, we achieved a state of hepatitis C eradication among kidney transplant recipients living in Qatar.

Several steps were taken to achieve HCV eradication in 2015. First, we identified all existing kidney transplant recipients with HCV infection. Second, we developed a protocol to screen all new kidney transplant recipients using HCV PCR. We also added HCV RNA testing as part of the first annual posttransplantation screening bundle for patients transplanted locally or abroad.

One of the main challenges highlighted in GHSS is drug availability and affordability. Some countries have been able to subsidize drug pricing and establish governmental reimbursement programs for HCV treatment.^15^, ^16^ We were able to overcome the expense of DAA therapy by getting the treatment subsidized by the government and establishing agreements with charity organizations to support those who could not afford DAA therapy. Initially, when the protocol was established in 2015, all DAA therapies available at our center were only sofosbuvir‐based, and three patients with eGFR < 30 ml/min could not be treated. In 2018, we were able to obtain non‐sofosbuvir‐based regimens such as elbasvir/grazoprevir; however, two of the three patients were already back to receiving hemodialysis by then. Our center has succeeded in the implementation of treatment for HCV in patients with advanced native CKD.^17^ Through this study, we were able to establish a successful pathway to treat all HCV‐infected kidney transplant recipients, and this is expected to open the doors for HCV‐positive to HCV‐negative deceased organ donation in the country in the near future.

Although significant treatment success has been achieved in non‐kidney transplant patients, the success rate for DAA therapy in kidney transplant recipients has varied from 89% to 100%.^11^, ^18^, ^19^, ^20^, ^21^, ^22^, ^23^, ^24^ Colombo et al.^25^ performed a phase 2 randomized trial involving kidney transplant recipients treated with DAA and reported SVR rates of 100% and 96% at 3 and 6 months, respectively. The present study yielded similar results despite the differences in race and HCV genotypes. Morales et al. reported an SVR rate of 96% in an African American recipient cohort, while Gutierrez et al. reported a rate of 89% in Hispanic recipients. Both studies included recipients with HCV genotype 1a. The association of race and the treatment response with socioeconomic or pathophysiologic factors needs further investigation.

The effectiveness of DAA in transplant recipients might also be associated with the type of immunosuppressant. In vivo studies showed that a combination of mammalian target of rapamycin (mTOR) with DAA increased HCV genotype 1b replication relative to that observed with CNI therapy, and this combination could account for treatment failure in kidney transplant recipients with HCV genotype 1b.^26^ All our recipients were receiving CNIs with DAA therapy; therefore, we could not evaluate the impact of mTOR on the effectiveness of DAA.

Despite the promising results of HCV eradication by DAA therapy in kidney transplant recipients, it remains unclear whether SVR will prevent long‐term complications of HCV infection, such as glomerulonephritis and posttransplant diabetes mellitus (PTDM).^27^ In our cohort, five patients developed PTDM before (*n* = 4) or during (*n* = 1) DAA therapy. We did not observe resolution of PTDM after an average follow‐up period of 2 years following DAA therapy.

We evaluated kidney function during the course of DAA therapy and found that three patients experienced AKI with a specific etiology: urinary tract infection, volume depletion, and tacrolimus toxicity (associated with macrolide antibiotics). All three patients showed recovery of their kidney function, which was similar to the baseline function at the end of treatment and after treatment completion. Moreover, acute rejection did not occur in any patient. Similar to our study, Kamar et al. reported no episodes of kidney allograft rejection, while Bhamidimarri et al. reported a 20% antibody‐mediated rejection rate in recipients treated with sofosbuvir and ledipasvir.^28^, ^29^ Morales et al.^24^ also reported an elevation of serum creatinine in 63% of 32 kidney transplant recipients treated with DAA; however, renal function improved in all recipients after treatment completion. AKI has also been reported in native kidneys with chronic disease, specifically HCV genotype 4 infection, or with the use of sofosbuvir/simeprevir.^30^, ^31^ These observations may be attributed to the retrospective study designs and the inclusion of advanced kidney disease in most of the studies to assess the cause of renal dysfunction during or after treatment. Of note, patients who completed the 1‐year follow‐up did not show a significant decline in eGFR; in fact, many recipients showed an improvement in kidney function. Aby et al.^27^ evaluated the progression of native CKD after DAA therapy and compared the findings with those for untreated patients; there was no significant difference in kidney function between the two groups at the 2‐year follow‐up.

DAA interaction with CNIs could have a significant impact on kidney function and rejection rates. Several studies have reported CNI dose adjustments in 18%–80% recipients during sofosbuvir‐based treatment.^11^, ^25^, ^32^, ^33^, ^34^, ^35^ Most of our patients had stable CNI levels before, during and after DAA therapy and did not require dose adjustment. However, one and two recipients required an increase and a decrease in the CNI dose, respectively. We observed a clinically significant tacrolimus trough increase from 9.2 ng/ml before DAA to 17 ng/ml immediately after simeprevir/sofosbuvir initiation and during the administration of azithromycin. Unlike some nonstructural protein (NS) 3 and 4A protease inhibitors such as grazoprevir, boceprevir, and telaprevir, simeprevir does not inhibit hepatic cytochrome P450 (CYP) 3A; accordingly, it is not considered to have drug‐drug interactions with tacrolimus.^36^ The clinically significant tacrolimus trough increase is most likely associated with azithromycin's inhibition of the hepatic CYP3A enzyme, although simeprevir may have contributed as it competes with CNI metabolism by the same CYP3A enzyme.^35^ In addition, simeprevir can inhibit P‐glycoprotein and intestinal CYP3A, both enzymes that contribute to the gastric clearance of CNI.^36^, ^37^ DAA may also reduce CNI levels, with the proposed mechanism being indirect activation of CYP during viral clearance. During HCV infection, proinflammatory cytokines inhibit CYP enzymes; however, the significant reduction in inflammation due to viral clearance during DAA therapy may increase CYP activity and reduce CNI levels.^38^

DAA therapy was tolerated well in our study, with mild side effects, mainly fatigue, and insomnia, reported in 30% cases. There were no cases of drug discontinuation caused by side effects. Overall, DAAs have been remarkably well‐tolerated by kidney transplant recipients. In the study of Colombo et al.^25^ adverse events, mainly headache, weakness, and fatigue, occurred in only 10% of cases. The percentage was higher in the MAGELLAN‐2 study of glecaprevir‐pibrentasvir in liver and kidney transplant recipients, where 22% of the study participants experienced fatigue and headache. The symptoms were serious in 8% of cases, and only one patient discontinued therapy early because of an adverse event.^22^

Our study strengths include the enrollment of patients from a single center that provides transplantation care for the entire country, the inclusion of different HCV genotypes, long‐term follow‐up duration, and low dropout rate. The limitations include the retrospective design, a small number of patients, and differences in the sofosbuvir regimens used.

In conclusion, in line with the WHO vision, we were able to achieve the eradication of HCV infection among kidney transplant recipients in Qatar by administering DAA therapy, which yielded a complete treatment response in all recipients infected with HCV. We foresee this to be a stepping‐stone toward the eradication of hepatitis C from the entire country.
